# Successful Impella 5.0 Catheter Removal in the Setting of Left Ventricular Thrombus With Use of the Sentinel Cerebral Protection Device

**DOI:** 10.1155/cric/3481920

**Published:** 2025-06-07

**Authors:** Anne Sandstrom, Kristen Errico, Debanshu Roy, Andrea J. Carpenter, Anand Prasad

**Affiliations:** ^1^Department of Cardiothoracic Surgery, UT Health San Antonio, San Antonio, Texas, USA; ^2^Department of Medicine, Divison of Cardiology, UT Health San Antonio, San Antonio, Texas, USA

## Abstract

Mechanical circulatory support devices, such as the Impella catheter (Abiomed, Danvers, Massachusetts), continue to become more commonplace in patients undergoing high-risk percutaneous coronary intervention (PCI) or those in cardiogenic shock. Thrombus in the left ventricle is a contraindication to Impella placement. Here, we present a patient with an anterior ST elevation myocardial infarction who underwent primary PCI with subsequent development of cardiogenic shock followed by Impella placement, who then later developed an LV thrombus. The Impella was removed after placement of a Sentinel cerebral protection device (Boston Scientific, Massachusetts). The left carotid filter of the Sentinel captured a thrombus fragment. The patient did not have any neurological compromise. This case represents the first report of actual capture of LV thrombus by a Sentinel system in this context. The case suggests the potential value of the Sentinel cerebral protection device to lower the risk of an embolic event during Impella removal in selective clinical scenarios.

## 1. Introduction

Mechanical circulatory support devices, such as Impella (Abiomed, Danvers, Massachusetts), have become increasingly utilized tools for patients with cardiogenic shock in the setting of ST elevation myocardial infarctions (STEMIs) [1]. Though the presence of a known thrombus is a contraindication for use of direct left ventricle to aorta support devices such as Impella [2], the development of LV thrombus in a patient with an Impella in place poses a difficult clinical dilemma to manage given the risk of maintaining support versus embolization. Here, we describe a the off-label use of the Sentinel cerebral protection device (Boston Scientific, Massachusetts) in a patient found to have an LV thrombus after an Impella 5.0 was placed for cardiogenic shock.

## 2. Case

A 60-year-old man presented to the emergency department with 3 days of chest pain and one episode of syncope. He was found to have an anterior STEMI. His clinical status rapidly worsened with the development of cardiogenic shock, requiring urgent intubation and multiple vasopressors. He was found to have a proximal left anterior descending (LAD) artery occlusion and a severe stenosis in the mid right coronary artery (RCA). He underwent percutaneous coronary intervention (PCI) to both the LAD and RCA with drug-eluting stents. Left ventricular (LV) end-diastolic pressure was 26 mm Hg, cardiac index of 1.15 L/min/m^2^, pulmonary artery (PA) wedge pressure of 18 mm Hg, central venous pressure (CVP) of 13 mm Hg, and PA oxygen saturation of 42%. The presence of diffuse atherosclerotic disease with vasoconstriction in his bilateral common femoral and external iliac arteries was prohibitive of the insertion of a femoral percutaneous transvalvular mechanical circulatory support device.

**Figure 1:**
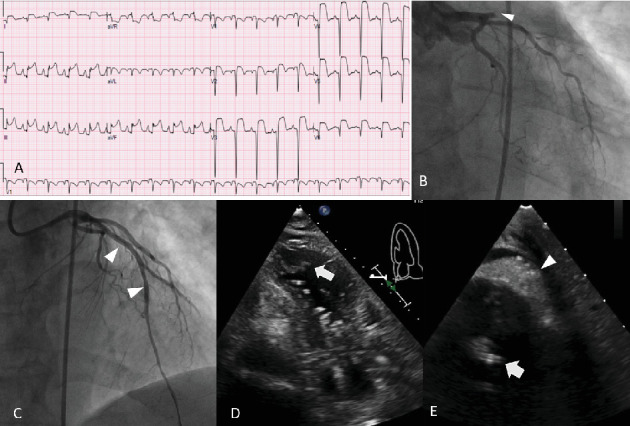
(A) ECG demonstrating ST segment elevation involving the anterior precordial and inferior leads. (B) Coronary angiogram with 100% proximal LAD occlusion (arrowhead). (C) Post PCI angiogram following revascularization of the LAD (arrowheads). (D) LV apical thrombus (arrow). (E) Epicardial thrombus secondary to LV apical wall rupture (arrowhead), LV apical thrombus in close proximity to Impella catheter (arrow).

Given his profound shock, an Impella 5.0 (Abiomed, Danvers, Massachusetts) catheter was placed via 10 mm Terumo Gelweave (Terumo, Scotland, United Kingdom) graft placed on the left axillary artery. Five hours after the procedure, the patient's hemodynamic status worsened with increased vasopressor requirements and decreased flow rates from the Impella. Bedside echocardiography was concerning for a possible LV free wall rupture and cardiac tamponade. An emergent bedside surgical pericardial window was created, leading to the removal of 200 ml of sanguineous drainage. Subsequently, the patient's blood pressure improved. Over the remaining 9 days, his hospital stay was complicated by shock liver, acute kidney injury, thrombocytopenia, sepsis, hemolytic anemia, and coagulopathy. He did eventually improve in his clinical status with intact neurocognitive function. A repeat echocardiogram was notable for a mobile apical thrombus in close proximity to the Impella catheter (Figure 1). Systemic anticoagulation resulted in some reduction in thrombus size, but without resolution. He was placed on a heparin drip with target anti-Xa levels of 0.3–0.7 IU/mL. LV ejection fraction improved from 30% to 40%, though his septum, mid inferior, and mid anterior walls remained hypokinetic with an aneurysmal apex.

**Figure 2:**
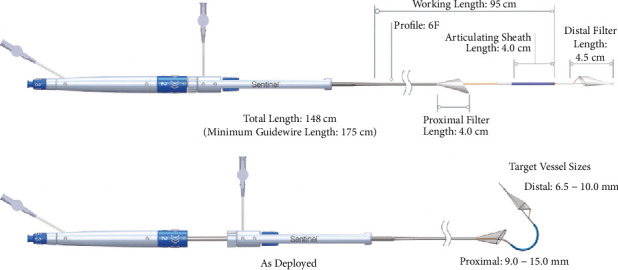
Illustration of the Sentinel device depicting the dual filters separated by an articulating sheath enabling placement into the innominate and left carotid arteries for cerebral embolic protection.

After weaning off vasopressor agents, the decision was made to remove the Impella catheter. There was concern over the potential for embolization of the thrombus during removal. Therefore, we elected to use the Sentinel cerebral protection device (Boston Scientific, Massachusetts) reduce the potential of cerebrovascular embolism (Figure 2).

**Figure 3:**
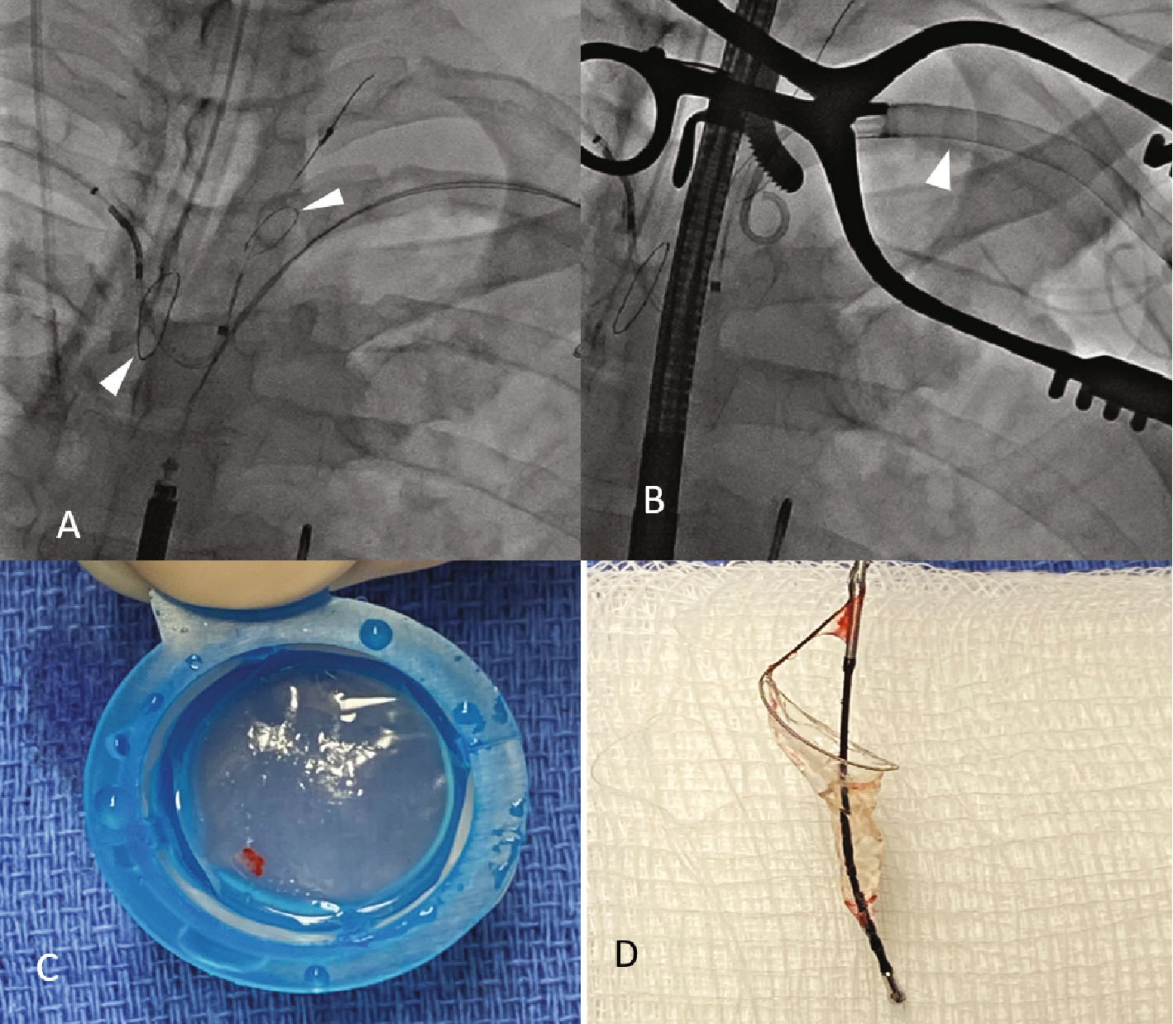
(A) Sentinel device; right brachiocephalic and left common carotid filters (arrowheads). (B) Impella catheter withdrawn into the subclavian artery during removal with Sentinel device in place (arrowhead). (C) An embolic particle captured during Impella removal. (D) Sentinel filter post Impella removal.

Using right radial access, the Sentinel device was positioned with the filters protecting the innominate and left common carotid arteries. The Impella was removed via the left axillary artery without interference with the sentinel device. The graft anastomosis was closed, followed by Sentinel removal. Upon inspection of the Sentinel, the left carotid filter had a visible red thrombus (Figure 3). Follow-up echocardiography immediately after the removal of the Impella catheter demonstrated persistence of the LV thrombus. A cardiac MRI was performed 1 week after device removal, which showed resolution of the thrombus. He was discharged on aspirin 81 mg daily, clopidogrel 75 mg daily, and warfarin with a goal INR of 2–3, with plans for 6 weeks of anticoagulation.

## 3. Discussion

Impella use for LV support has risen significantly in the past decade, with cardiogenic shock related to STEMI being the most common indication [1]. Mean duration of Impella use is typically 1–4 days; however, extended use in cases of shock or bridge to further support or recovery has been reported [2, 3]. The overall in-hospital incidence of LV thrombus in STEMI patients is reported to be < 1% [4]. Proinflammatory and hypercoagulable states coupled with impaired regional wall motion after an anterior MI have been associated with LV thrombus formation [5]. Intracardiac sources of thromboembolism, whether related to mechanical circulatory support device catheters/pumps or independent of them, pose a significant risk of stroke or peripheral embolism.

**Figure 4:**
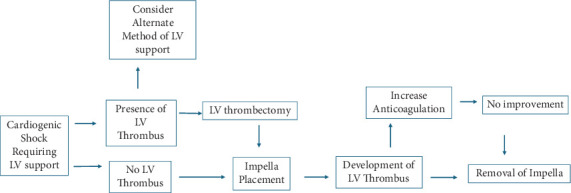
Proposed treatment strategy for patients with LV thrombus requiring Impella support.

Ischemic strokes attributed to Impella pump use have been reported [6]. Recent reported rates of in-hospital stroke in cardiogenic shock patients requiring Impella support have been as high as 6% [1]. However, the etiology of these events is unclear and likely variable. Thrombus formation on the Impella device is rare with adequate use of anticoagulation. The presence of a known LV thrombus is a contraindication to Impella catheter placement. However, LV thrombectomy followed by successful Impella placement has been described [7]. The development of LV thrombus in a patient with an Impella already in place poses a challenge for management. The risk-benefit ratio of maintaining support versus the risk of embolization should be made on a case-by-case basis. A trial of increased anticoagulation intensity may be considered. The treatment strategy for patients with LV thrombus requiring impella support is summarized in Figure 4. Thrombectomy using a Penumbra lightning catheter or ONO retrieval device could be considered; however, neither such device was available to us at the time of this case. This could be an option to employ, but one would need to consider the feasibility of manipulating this device across the aortic valve alongside the Impella and also consider the risks of entering the left ventricle, including arrythmia or perforation. A clear strategy for safe removal of Impella catheters in the presence of known LV thrombus has not been established. The present case suggests that the use of cerebral protection may be of value. A similar approach was taken by Sachedina et al. and Yeoh et al. [8, 9]. Sachedina et al. described a 1.2-cm adherent clot at the LV apex which formed after Impella CP use for 5 days. Yeoh et al. described a laminar thrombus in the LV in the context of high-risk PCI and TAVR with Impella support. Our case is unique in that we demonstrate visible capture of thrombus by the Sentinel device.

## 4. Conclusion

LV thrombus complicates approximately 1% of anterior STEMI cases. LV thrombus associated with/in proximity to an Impella catheter poses a unique challenge, especially when considering safe removal of the catheter. Off-label use of the Sentinel cerebral protection device can be considered in carefully selected patients.

## Data Availability

All data generated or analyzed during this study are included in this published article.
